# EXTENDING A HEALTHY LIFESPAN WITH 3-HYDROXYANTHRANILIC ACID

**DOI:** 10.1093/geroni/igac059.2662

**Published:** 2022-12-20

**Authors:** George Sutphin, Hope Dang, Raul Castro-Portuguez, Luis Espejo, Sam Freitas, Jeremy Meyers

**Affiliations:** University of Arizona, Tucson, Arizona, United States; University of Arizona, Tucson, Arizona, United States; University of Arizona, Tucson, Arizona, United States; University of Arizona, Tucson, Arizona, United States; University of Arizona, Tucson, Arizona, United States; University of Washington, Tucson, Arizona, United States

## Abstract

Metabolism of tryptophan by the kynurenine pathway is increasingly linked to aging. Kynurenine pathway enzymes and metabolites influence a range of molecular processes critical to healthy aging, including regulation of inflammatory and immune responses, cellular redox homeostasis, and energy production. Aberrant kynurenine metabolism occurs during normal aging and is implicated in many age-associated pathologies including chronic inflammation, atherosclerosis, neurodegeneration, and cancer. We and others previously identified three kynurenine pathway genes—kynu-1, tdo-2, and acsd-1—for which decreasing expression extends lifespan in invertebrates. More recently we discovered that knockdown of haao-1, a fourth kynurenine pathway gene encoding the enzyme 3-hydroxyanthranilic acid dioxygenase (HAAO), extends lifespan by ~30% and delays age-associated health decline in Caenorhabditis elegans. Lifespan extension is mediated by increased physiological levels of the HAAO substrate 3-hydroxyanthranilic acid (3HAA). Aging mice fed a diet supplemented with 3HAA are similarly long-lived. The mechanism of action liking 3HAA to aging is complex and partially overlaps with multiple pathways previously implicated in aging. We recently identified activation of the Nrf2/SKN-1 oxidative stress response and alterations to iron homeostasis as key players in the benefits 3HAA. Ongoing work explores the relationship between 3HAA, Nrf2/SKN-1, and iron in C. elegans and mammalian aging, age-associated immune decline, and cancer. This works provides a foundation for detailed examination of the molecular mechanisms underlying the benefits of 3HAA, and how these mechanisms interact with other anti-aging interventions. We anticipate that these findings will bolster growing interest in developing pharmacological strategies to target tryptophan metabolism to improve health aging.

